# Chronic Dysentery Topically Treated

**Published:** 1877-03-20

**Authors:** T. Wertz

**Affiliations:** Jasper, Ind.


### CHRONIC DYSENTERY TOPICALLY TREATED.

A Report of a Case read before the Du bois Co. Medical Society, Nov. 17th, 1876, by T. WERTZ, Jasper, Ind.

I saw Mrs. R———, for the first time, Feb. 28, 1876, and found her suffering with chronic dysentery of six months standing. She was at that time in a very critical condition, very frail, and reduced in flesh to a mere skeleton. She had a great aversion for food, and usually vomited whatever eaten, a short time after swallowing it. The pulse 120 per minute, weak and very compressible; the skin, shrunken, and bathed in a cold, clamy sweat. There were from fifteen to twenty actions from the bowels, in the twenty-four hours, consisting of mucus, blood and pus. A tormenting tenesmus, with scalding of the urine, made life a perpetual torment, and deprived the patient almost wholly of sleep.

The patient had been treated by a number of physicians, and I was satisfied all had been done by internal medication that offered any hope of a cure. I at once proposed the topical plan of treatment, and the patient being very tired and nervous refused to submit to it. She then had for awhile bismuth and morphine without any benefit. There being a desire for something sour, Hope's mixture was ordered, which seemed to benefit her for a time, but she soon relapsed to her old condition and as a last resort, consented with much fear and trepidation to submit to local treatment.

March the 21st—assisted by Dr. Crook the patient was put under the influence of chloroform, placed in the left lateral position, and the sphincter ani was fully dilated by stretching. The rectum was then thoroughly cleansed by means of a cotton swab and water. (Dr. Themin, of New York, uses a glass tube bent upon itself, and attached to a Davidson's Syringe for the purpose). A duck-bill speculum, previously well oiled, was slipped into the rectum and held by an assistant in the position usual for vaginal inspection, and with a depressor on the anterior wall of the rectum, a good view was obtained for some distance up, a further view being obstructed by the pressure of the gravid uterus, the patient being advanced to the fifth month of pregnancy. The mucous membrane of the lower bowel was inflamed, much thickened, and studded with numerous ulcers, of various sizes, and bleeding when touched. The rectum being well cleaned out, a solution of nitrate of silver, two drachms to the ounce of water, was thorougly applied to the parts. She soon rallied from the effects of the chloroform, and complained of pain in the part cauterized, which was readily relieved by an opiate, after which the patient had a sound sleep for ten consecutive hours. This was the first good sound sleep the patient had had for several months.

Sleeplessness, I believe, is always a prominent symptom in chronic dysentery. The frequent evacuations, and spasms of the sphincter muscles and lower bowel, produce a reflex excitability of the nervous system incompatible with repose.

It required about four days for the paralyzed sphincter to regain sufficient tonicity to retain the liquid contents of the bowel. After this the evacuations were reduced in frequency and contained less blood.

Six days from the time of the first application, assisted by Dr. Crooke, the patient was carried to the operation table, placed under the influence of chloroform, and the nitrate of silver applied as before, except, that it was carried higher up the bowel into the sigmoid flexure. This portion of the bowel is usually the seat of numerous ulcers, and care should be taken to carry the caustic well up into the sigmoid flexure of the colon. After the second application the improvement in the patient's condition was rapid and steady. The actions from the bowels were reduced to four or five per day, and assumed more the characteristics of healthy discharges.

A milk diet was ordered from the first, but from the long use of a slop diet, the patient had become disgusted with it, and on her own responsibility, took a full range of a solid dietary, such as fish, ham, fresh beef, etc., all of which she now began to relish. As the stomach retained and digested the above mentioned food, no restrictions were laid on her choice of it. She rapidly gained in flesh and strength, and in a few weeks was able to leave her bed and walk about the house, a privilege she had not been able to enjoy for a long time, The only additional treatment the patient received during this time, was an opiate and bismuth powder for the first three days, which was dis continued at her request.

July 27, Mrs. R——, was delivered after a short labor. The child though tolerably well developed, showed many evidences of the feeble nourishment it had sustained during the greater portion of intra-uterine gestation. For two months previous to delivery Mrs. R—— had fully regained her health and passed through her confinement very rapidly.

This case testifies very strongly in favor of the topical method in treating those cases. In view of the extreme emaciation and pregnant condition of this patient, it affords very gratifying evidence of the power of therapeutical agents in aiding the system in casting off disease.—*Cin. Lancet & Observer.*

